# Stepping From Modeling Cancer Plasticity to the Philosophy of Cancer

**DOI:** 10.3389/fgene.2020.579738

**Published:** 2020-11-19

**Authors:** Jean Clairambault

**Affiliations:** ^1^Laboratoire Jacques-Louis Lions, BC 187, Sorbonne Université, Paris, France; ^2^Inria, Paris, France

**Keywords:** philosophy, therapeutics, evolution, multicellularity, cancer

## 1. Introduction

Coherent multicellular organisms are not only cohesive from a spatial and anatomical point of view but also coherent from the phenotypic and cell-functional point of view of compatibility, cooperativity and division of tasks between cells and tissues. This is mandatory to make possible the achievement of a stable, functional, and reproductive whole.

Leaving aside the possibility of spontaneous “emergence of order from chaos,” this article proposes a simpler hypothesis of a system of communication ways between trees of differentiation, relying on the control of transcription factors that determine differentiation: “the cohesion watch.” These are considered a part of the immune system, whose armed force is the immune response, innate as well as adaptive, humoral and cellular, but is not the whole of it. Indeed, this paper poses that the immune system is the coordinator of the unity of the organism. Within the immune system in this extended vision that is thus more general than the immune response, the cohesion watch is in charge of the control of compatibilities and cooperation between the anatomical and the phenotypic/cell-functional systems as well as within each of these systems. It is a mandatory component of multicellularity that ultimately leads to an anatomically cohesive and functionally coherent organism.

The immune system in this extended sense should thus comprise the following: (a) the equivalent in all Metazoans of the major histocompatibility complex (MHC) of jawed vertebrates, in charge of characterizing all cells of a given individual within its species (this article postulates the existence in all Metazoans of a coding system analogous to the MHC of jawed vertebrates that is present in all its forerunners in animal evolution); (b) the immune response; and (c) the cohesion watch. The latter is here assumed to be a complementary histocompatibility complex in charge of the following:

- driving indifferently (i.e., in an equal way) in each organism-to-be in a given species the body plan (or Bauplan, described with regulatory mechanisms in Davidson et al., 1995 for bilaterians and for older animals in evolution in Müller et al., 2004) by launching morphogens during embryonic development;
- simultaneous with the indifference, guiding from pluripotent stem cells attached to the body plan the development of the trees of cell differentiation; and
- establishing between the trees and twigs of differentiation that stem from the body plan acellular compatibility regulatory mechanism, the main compatibility task of the cohesion watch.

These trees, in the Waddington view (inverse, in its three-dimensional presentation, of the tree expansion metaphor), are none else than the epigenetic landscapes, and they are controlled by transcription factors and epigenetic enzymes. All differentiations lead terminally to mature cell types, between 200, and say, 400 (according to various evaluations) (Jacob, 1977) in the human species but only 20 in the sponges Porifera (Müller, 2001). They are in any event of a fixed number for every organism in a given species. Inscribed, much like the body plan and the functionality differentiation trees, in the genome of each cell, this cohesion watch should manifest itself materially during development as a net of communication between and within differentiation trees. At the chromatin level, it should control the non-expression (in closed-state chromatin), expression or repression (in open-state chromatin) of genes at nodes in the cell differentiation trees, and it should also control the stability of the body plan.

## 2. Modeling Plasticity in Cancer Cell Populations

In a series of papers starting in 2013, a team of mathematicians (the author included) at Laboratoire Jacques-Louis Lions, Sorbonne University, Paris, and some followers elsewhere were initially stimulated by an article published in 2010 (Sharma et al., 2010) that reported reversible drug resistance in a cancer cell culture. The culture, exposed to massive doses of drugs, developed in sparsely distributed resistant subpopulations (named persisters), and such resistance, shown to be of a non-genetic nature, was completely reversed when the drug was withdrawn from the culture. Driven by this biological observation of reversible resistance in cancer cells, we tackled the question of understanding and predicting the dynamics of these cancer cell populations by using mathematical models. The behavior of these highly *plastic* cell populations was relevantly described by phenotype-structured partial differential equations. In these equations, the structuring variable, i.e., the parameter-like one that codes for the biological variability of interest, is assumed to store the *heterogeneity* of the cell population with respect to the expression of drug resistance. It was chosen to be a positive real variable representing the expression of a resistance phenotype *continuously* from 0 (totally sensitive) to 1 (totally resistant) (Perthame, 2007, 2015; Lavi et al., 2013; Lorz et al., 2013, 2015; Chisholm et al., 2015, 2016a,b; Lorenzi et al., 2016; Almeida et al., 2018, 2019; Cho and Levy, 2018a,b; Clairambault, 2019; Clairambault and Pouchol, 2019; Nguyen et al., 2019).

These models, intended to represent the effects of a cancer treatment on cell populations, and ultimately on patients, with the aim to overcome their capacities of resistance induced by the treatment itself, naturally give rise to the proposal of theoretically optimized therapeutic strategies. Such strategies, which have recently been the object of active research (reviewed in Jarrett et al., 2020) aim to contain or eradicate cancer growth, avoiding the two major pitfalls of treatments in clinical oncology, namely unwanted toxic side effects in healthy cell populations and the emergence of resistance in cancer populations (Pouchol, 2018; Pouchol et al., 2018). These strategies, which are still theoretical, may be too recent to be widely accepted by oncologists as plausibly efficacious and challenged by preliminary experiments, in Petri dishes or in laboratory rodents. In the meantime, many questions arise about the nature of plasticity in cancer cells and cancer cell populations that underly them (reviewed in Shen and Clairambault, 2020). Cancer is a disease of multicellular organisms that are normally functionally constituted of terminally and irreversibly differentiated cells. We have made progress in understanding the causes and the mechanisms of the reversion of differentiations that make cancer cells so plastic. Are we are able to quickly adapt their phenotypes to a changing environment, such as deadly drug pressure, while healthy cells cannot?

## 3. Questions About Multicellularity and Cancer

Some motivations for the interest of stepping away from such therapeutically oriented models of drug resistance in cancer cell populations, and thus of plasticity in cancer, to more general considerations can be seen as arising from observed facts. Such questions are most of the time dodged, likely being perceived as too complex to be solved by specialists of one domain only, in the field of cancer biology:

- Cancer can be found throughout the animal kingdom (Aktipis et al., 2015) and beyond, but plants, however, are not lethally affected by it (Doonan and Sablowski, 2010); investigating the earliest stages of multicellularity in animals (Müller, 2001; Müller and Müller, 2003; Müller et al., 2004), i.e., searching for its failures, may therefore be a natural way to understand how some somatic cells become cheaters to their established multicellular community.
- The genes that are altered in cancers are the same that serve a multicellularity design (Domazet-Lošo and Tautz, 2008, 2010; Davies and Lineweaver, 2011; Vincent, 2011a,b; Lineweaver et al., 2014); can we methodically collect these genes?
- What defines identical organisms? A “self” conserved during sequences of differentiation that in humans developed from the first embryonic cell to the about “200 terminally differentiated cell types”? Interesting answers are suggested in different works dealing with the philosophy of biology or the “philosophy of cancer” (books by Pradeu, 2012, 2019; Bertolaso, 2016; Laplane, 2016; Plutynski, 2018, and others).
- Can we envision the immune system not as limited to the immune response to pathogens and abnormal host cells but rather as a law of cohesion for the whole organism construction?
- Would not the immune response be in this extended vision of the immune system only its “sword arm,” a police patrol and pale reflection of the law itself, whereas a hidden part of the immune system would be the “spirit of laws” (analogous, *mutatis mutandis*, to Rousseau's unwritten social contract in human societies)?
- What holds together, normally without conflict, the cell types? Is it not something that governs development from the beginning—something more than what the immune system uses when it recognizes as non-self (foe rather than friend) a cancer cell?
- Is there a complementary relationship between the maintenance of such coherence and the major histocompatibility complex (MHC), or rather its likely forerunners in non-vertebrates, yielding early adaptive immunity?
- What is the primary function of the immune system if not to ensure organism cohesion (of tissues), and how does such coherence (of signals) operate? If it is so, what is the impact of this (extended, i.e., going beyond the classical cellular and humoral immune response and earlier than it in the construction of multicellularity) version of the immune system on cell differentiation?
- Is the immune system the “glue” (Pradeu, 2012, 2019) that holds together the cells and functions of the multicellular organisms we all are constituted of together until such cohesion/coherence is altered in cancer?

## 4. The Atavistic Theory of Cancer

### 4.1. The Theory in a Nutshell

According to the atavistic theory, cancer is a local regression of a stable multicellular organism (Metazoa 2.0) to an incoherent state of a cell colony (Metazoa 1.0), non-existent as an evolution entity since it is not stable and is incapable of reproducing itself. This state is supposed to have predated the transition toward established stability that defines a stable and reproductive multicellular organism as a Darwinian selection unit. This point of view has been proposed at least in 1996 (Israel, 1996), and likely earlier, but was popularized in 2011 by Davies, Lineweaver, and Vincent (Davies and Lineweaver, 2011; Vincent, 2011a,b; Lineweaver et al., 2014; Thomas et al., 2017) and then examined from the point of view of the history of genes (Domazet-Lošo and Tautz, 2008, 2010; Wu et al., 2015; Bussey et al., 2017; Cisneros et al., 2017; Trigos et al., 2017, 2018, 2019). The atavistic theory of cancer has also recently been compared (Lineweaver et al., 2020) with the dominating (among cancer biologists) somatic mutation theory (SMT, that is more often compared with tissue organizational theory, TOFT) (Soto and Sonnenschein, 2004; Sonnenschein and Soto, 2016), and popularized in review articles (Goldman et al., 2017a,b). It poses the question of transition to multicellularity, for which we have to elaborate a plausible scenario, not sketched by the abovementioned authors.

### 4.2. Stage 0, aka Metazoa, The β Version

At this elementary stage of multicellularity, where proliferation limited by apoptosis is the only possible fate for cells (note that the emergence of apoptosis in evolution is studied in depth in Koonin and Aravind, 2002), they stick together in the ocean thanks to a form of collagen glue. Note that the existence of collagen implies enough availability of oxygen in the oceans, which dates this episode back to at least −850 million years. These cells are then able to exchange information, either by paracrine communication or by gap junctions (Trosko, 1987, 2007, 2016), through innexins present, e.g., in Hydra, rather than through connexins (Alexopoulos et al., 2004) or others (Mitchell and Nichols, 2019). Gap junctions allow cells to exchange molecules that can be toxic, such as oxygen, which can be indeed toxic before endosymbiosis of mitochondria in eucaryotes. With regards to the properties of cells at this stage, we assume only proliferation and its dual property, apoptosis, to be both influenced by environmental factors. We also assume a friend-or-foe recognition system to be present in each cell and they can use intercellular communication (paracrine or via gap junctions). Now, what should be the use of such a system if it would not react when a message testing an external intruder registers a foe, i.e., that we are under attack? Assuming no specialization (i.e., no differentiation and no division of work) at this stage, collective fright, fight or flight may be represented by a hedgehog-like attitude, the secretion of toxins in the environment, and collective movement without individual or semi-collective cell motility, respectively. Note that the latter is shown by tumor spheres with inverted polarity, TSIPs, which are moving hedgehogs or urchins (Zajac et al., 2018) encountered in breast and colorectal cancer cell populations. The genome of each of these cells has evolved to grant them such properties, making them able to resist UV radiation, acidity, cytotoxic molecules, hypoxia (after the endosymbiosis of mitochondria in the case of animal cells). A bond between them must exist that defines each of them as a member of a colony—a kind of *self* that controls proper cell division. This *self* and the friend-or-foe recognition system are assumed to be remote ancestors of the major histocompatibility complex (MHC, the common law in jawed vertebrates) and of the humoral immune response of vertebrates, immunoglobulins.

### 4.3. Stage 1, aka Metazoa 1.0

In the following stage, under the pressure of successive hostile attacks from the environment, begins the *reversible* division of work, i.e., differentiation of subpopulations of cells to allow them to perform specialized tasks, and this does not involve the whole cell population in all the tasks. According to Maynard Keynes and Szathmáry (1995), the first of such specializations could be the constitution of the germen (germinal cells), in charge of propagating the common genome, as opposed to the stroma (stromal cells), in charge of protecting and preserving the germen by all possible means of further specialization, e.g., motility, production of secretions, fast communications, etc. Differentiations producing division of work then appear, and they occur according to molecular determinants inscribed in the DNA, contacts between neighboring cells, and according to physical laws of soft matter that determine them in 3D space (Fleury, 2013). These differentiations are, however, very labile, i.e., reversible; otherwise said, the cells at Stage 1 are endowed with high plasticity with respect to their phenotypes.

Due to such plasticity, which prevents coherent construction of an organized cell colony that could be divided in cooperating subpopulations, no stable structure can emerge at this stage. The sketch of the immune system of Stage 1 has not evolved. On the contrary, something of the emerging self may be lost, as cell divisions may be futile, with junk DNA (the common law is easily trespassed and ignored) and existence of monster or non-viable cells. No working immune system leading to a stable coherent whole can exist in such cell populations. A Stage 1 cell colony is, according to the atavistic hypothesis of cancer (Davies and Lineweaver, 2011) characteristic of cancer cell populations, found in tumors. Many properties available in tumors, such as high individual plasticity, adaptability to external insults, loss of the common self (as all cells are potential defectors—cheaters—with respect to the poor common law of Stage 1) and no regulation of proliferation nor of differentiation, are present. Proliferation (fecundity) and apoptosis are now completed with differentiability and de-differentiability, i.e., extreme cell plasticity. Cooperation between subpopulations may exist (Tabassum and Polyak, 2015) though not on a perennial nor consistent basis. From a metaphoric Waddington landscape point of view (Waddington, 1957; Huang et al., 2007; Huang, 2011, 2013), the scenery is flat or with unpredictably changing slopes. What can you build with plasticine bricks?

At this stage, the colony of cells is a soft and moving mixed cellular and acellular “soup.” To achieve the transition from it to stable multicellularity (Newman, 2016), one can imagine that, if all elements in the genetic roadmap are present at least in some of these cells—in particular if sexed reproduction is also already active, such as in yeast cells—then physical laws of soft matter would drive this soup to a more consistent material. Indeed, mathematical natural gradient dynamics and singularity unfolding (Thom, 1972; Fleury, 2013) can be represented by chemical reaction-diffusion equations (Turing, 1952) at work in morphogen gradient-guided embryology processes. Many attempts to multicellularity may have occurred (and evolutionary biologists tell us that there have been may failed attempts) until a stable cohesion watch (maybe established, e.g., on paracrine or Delta/Notch communication or through gap junctions) can actually emerge and stabilize the structure of the plan. Then any fecundation that launches the division of a fertilized egg can be successful to yield a multicellular organism.

### 4.4. Stage 2, aka Metazoa 2.0

At Stage 2, an organizational principle emerges from the eddying chaos of Metazoa 1.0 and takes control of differentiations and proliferation. The common law is respected by all cells of the colony, and it can defend itself as a whole entity against attacks and can now inscribe itself in the fate of Darwinian evolution, maintained as a coherent ensemble by a functional immune system and a nerve communication system. The primitive Urmetazoa, as described in Müller (2001), Müller and Müller (2003), Müller et al. (2004), and Srivastastava et al. (2010), may have been a kind of sponge much like Porifera. The multicellularity gene toolkit of Metazoa 2.0 (Davies and Lineweaver, 2011) appeared at this stage, quite early and long before the Cambrian explosion, close to a date around −800 million years (Müller et al., 2004). What is this new collection of genes made of and how has it been hierarchically organized with respect to preexisting genes of unicellularity (e.g., cell cycle control)? What is the common law that defines an individual as any representative of its species (between-species distinction)? What defines a particular individual within its species (within-species distinction)? These questions ought to be documented to better understand what support this point of view may bring to the documentation of the idea of a hierarchical organization of the genome.

The immune system is now not only in charge of friend-or-foe recognition and defense of the colony when it is under attack, but it has, more importantly, emerged as a centralizer principle under the form of a chip present in every cell, ensuring the consistency of the whole construction. This common “law” is inscribed in the genome of each cell. Cheater cells may exist as in every organized society; however, they are sensed by a specialized subpopulation of cells (the police or immune cells) endowed with the mission to contain or destroy them. From a molecular point of view, repeat regions in the genome [in particular LINE-1 (Guler et al., 2017) in connection with the interferon pathway] could be responsible for such sensing. From the metaphoric Waddington viewpoint (Waddington, 1957; Huang et al., 2007; Huang, 2011, 2013), an irreversible differentiation potential (Zhou et al., 2012, 2018) is now present. With regards to the material construction of a stable organism, bricks and enamel are ready to be cooked in an oven, and perennial Assyrian palaces can now be built. What such virtual ovens consist of that will stabilize the multicellular organism during development we do not know; we can only suppose that some genes are silenced throughout this stabilization process.

Yet the fact remains that within the developmental stage of this construction, plasticity (reversibility with respect to a differentiation potential) is necessarily present for a limited time. This is the time of embryological development. After this time, the so-called Yamanaka genes (Takahashi and Yamanaka, 2006) Oct3/4, Sox2, c-Myc, and Klf4, that can reverse differentiation to produce induced pluripotent stem cells (iPSCs) are normally silenced (they can be revived in cancer, disease in which cells have not been properly “cooked” by gene silencing at some differentiation stage). Nevertheless, we know that some Metazoa, like the salamander (or axolotl), are able to locally go back to this developmental stage and regrow a tail or even a limb when it has been severed from the body.

The molecular level at which such control on differentiations is exerted is likely the level of the chromatin, where epigenetic enzymes, themselves coded by epigenetic genes, exert their control on the expression of genes, possibly by controlling transcription factors. The sequence of mutations observed in acute myeloid leukemia (AML), in evolutionary time firstly on epigenetic control genes, then on transcription and differentiation factors, and only finally on genes of proliferation (Hirsch et al., 2016), seems to recapitulate in reverse order the sequence of stages proposed here. One can suspect that a hierarchical relationship, such as that mentioned above about repeat sequences and the immune system, exists among control of gene expression at the chromatin level. Where a repository of an MHC-like common law, i.e., of marks defining not a particular individual but something common to all individuals of a given species and control of differentiations by the immune system, could exist is an open question. Indeed, such epigenetic/immune control of differentiations is not documented but is likely to exist.

To sum up this stage, there is persistent division of work since it appeared at Stage 1 already, but now it is consistently organized as *irreversible* differentiation. This constitutes a new fate (added to proliferation, apoptosis and senescence) in the physiological cellular life in each cell under the control of the cohesion watch during development. Later, added to the cohesion watch, specialized populations of cells, the immune patrol police, have appeared when the organism has been completely built. They oversee surveillance and the containment or destruction of trespassers. The cell colony, now a Metazoan 2.0 endowed with a functional immune system and able to reproduce itself, can successfully go through the tinkering (Jacob, 1977) of Darwinian evolution from sponges to vertebrates. However, in case of malfunction of any of its parts, due to malfunction of the immune control (insufficient control) on its differentiation fate, this part is likely to revert to Stage 1, aka Metazoa 1.0, according to the atavistic hypothesis of cancer (Davies and Lineweaver, 2011). Conversely, when the police patrols (lymphocytes and macrophages) overreact, wrongly interpreting normal signals as trespasses, this may lead to allergies and auto-immune diseases.

## 5. What Is a Functional Multicellular Organism?

### 5.1. A Borromean System Responsible for the Emergence of Metazoa

The construction of the mind proposed now as common to all individuals in a species thus consists of the following:

(a) a base for the construction—the anatomical system, sets of genes in charge of the spatial embryological development, i.e., the 3D body plan (Müller et al., 2004; Amundson, 2005), and tissue/organ morphogenesis included;
(b) attached on this base to points that are virtual tissue-specific stem cells, domains of differentiation stemming as tree-like structures (inverted Waddington landscapes) of functionalities, i.e., sets of nodes of differentiations specific of a given functionality, e.g., in vertebrates, digestion, circulation and body covering, that in particular will yield the up to 200–400 functional human cell types (Jacob, 1977); and
(c) a hypothesized “cohesion watch” and complementary histocompatibility control system, which is a net made of connections—nervous, hormonal or by cell-to-cell contact—between and within the functionality trees in charge of controlling compatibilities and cooperation within each of the two systems and between the two of them, to achieve a cohesive and coherent multicellular system.

The whole construction should possess the characteristics of a Borromean system (endowed with the Brunnian property: removal of any one component unlinks the entire system) of length 3 (Chichak et al., 2004; Baas et al., 2015). Each subsystem exists independently of the other two, though no common sense can be obtained, in order to achieve the coherent design of a multicellular organism without the simultaneous participation of all three to the design. Furthermore, if any of them dissolves in the environment or fails its task, the other two may continue their separate existences, though this does not lead to a viable organism or else an impaired one. For instance, in the case of failure of control on the human body plan only, and in increasing order of gravity, we could see possible limb agenesis, partial rachischisis (spina bifida), and anencephaly except for the latter case in viable organisms.

The case of cancer, a disease specific of multicellular organisms, and in as much as it may destroy the whole organism, specific of animals [aka Metazoa, characterized by heterotrophicity among multicellular organisms; cancer exists in plants but remains localized and is not lethal (Doonan and Sablowski, 2010)], is the result of primary partial (local) failure of the compatibility control system (the cohesion watch) on the phenotypic coherence of the organism. In cancer, the body plan (in an extended sense, i.e., 3D anatomical shape and functional organ morphogenesis) is usually respected, but failure of control on differentiations (at the level of trees or inverted Waddington landscapes) gradually leads to incoherence in the cooperation tasks (improper division of work) between tissues and organs. Then the natural history of the disease leads to dissolution of the organism as a whole [de-unification of the individual, as Pradeu (2019) writes].

### 5.2. In More Detail, Why Is It a Borromean Structure?

Should the cohesion watch be firmly attached to the body plan but with missing places there for the trees of functionalities relying on phenotypic differentiation, this could (however unlikely in reality) lead to void shapes that one can figure as development stopped at different embryonic stages, e.g., gastrulation [in triploblastic animals (Seilacher et al., 1998; Martindale et al., 2004)] or neurulation (in vertebrates). If, conversely, it controls all trees responsible for cell-functional phenotypes, when all necessarily cell specializations have been achieved, but the body plan is loose (not cohesive), then division of work is there, and everything is ready for the emergence of a virtual Metazoan except that it cannot be embodied in a stable spatial and functional structure and thus cannot exist. Furthermore, the cohesion watch, an epigenetically controlled non-cellular system of intercellular communication controlling differentiations, must make these differentiations irreversible to yield a stable multicellular organism. Before its appearance in evolution, differentiations were partially or completely reversible, which was in particular useful to making the whole construction able to mobilize enough cells in the colony to face an incoming external aggression. This might be by motility and by specialization into protecting cells, precursors of immune cells, facing them by fight, flight, or fright. In the metaphor of the Waddington landscape, such irreversibility is ensured by the establishment of high epigenetic barriers that prevent de-differentiation or transdifferentiation. Indeed, evolution cannot build anything perennial on moving ground (non-moving meaning here a permanent spatially and functionally organized support within which cell subpopulations can cooperate to establish an individual able to feed on its environment), avoid destruction from it and secure its reproduction.

The cellular immune system cannot appear out of thin air but could emerge from a specialization from a primitive immune-like cell type in the initial cell colony, yielding cells and signaling molecules able to recognize both the MHC, or rather its forerunners in evolution, by tagging an individual in a given multicellular species. It will also be able to recognize common markers, tagging the species, in any cell of the colony. The next stage would be to validate them as faithful elements of the ensemble or else to destroy them or reject them from the cell colony by making use of an armed force, the (cellular and humoral) immune response. These specialized immune cells should then take control of all other cells of both the anatomical development system (the materially established body plan) and of the epigenetic system of differentiations rendered irreversible by the cohesion watch to then emerge during early embryogenesis a truly stable Metazoan. The article envisions the cohesion watch as a set of intercellular communications assumed to be present in all cells of a Metazoan 2.0 (including the emerging immune cells) under the form of a program that is the basis of the “common law” of the species. Such a dual event, preexisting cohesion watch in all cells—the common law—and enforcement of the cohesion by the materially constituted cohesion law and by the emerging immune cells during embryogenesis—the sword arm or police—is highly evocative of the constitution of an emergent Borromean system. Before its emergence, it can only exist as tumor-like Davies's and Lineweaver's Metazoa 1.0, and after it is constituted, it exists as a cohesive and stable Metazoan 2.0 (a true Metazoan).

### 5.3. The Basic Anatomic System: The Body Plan in Development

The structure of the body plan (Davidson et al., 1995; Müller et al., 2004) is not easily defined, as it has evolved along with the evolution of species. However, one might define it, independently of the animal species under consideration, as the anatomically based collection of all organism functionalities. Well-known by embryologists for quite a long time, long before the emergence of the study of genetics and the knowledge of the roles of body plan genes, the embryological development of animals has been described from the blastula stage (a 2D sphere made of undifferentiated cells) until the constitution of forms that depend on the species. These forms resort to diploblastism (two layers: the endoderm and ectoderm) in elementary Metazoa, such as placozoa, ctenophora or cnidarians, and later triploblastism (three layers covering a 2D-sphere: the endoderm, ectoderm, and between them, mesoderm) in all others. Triploblastic animals appeared between 1 billion years and 600 million years ago and were later structured by a hard skeleton during the Cambrian explosion, which began 541 million years ago and lasting for about 13–25 million years. In triploblastic animals, particularly in vertebrates, gastrulation and neurulation are dynamic phenomena in which cells follow flows that will constitute their anatomic structures. They have recently been described from a physicist's point of view by Fleury (2013) and from a mathematician's point of view, much earlier, by Thom (1972). No genes are present in the points of view of these authors. However, the explanation of the formation of embryological layers due to the dynamics of morphogen gradients, firstly predicted by Turing (1952), now identified as, e.g., Wnt, and controlled by, e.g., Hox, is presently the norm—all the more so as knock-out embryos (mice and flies) for these genes are currently documented to help us understand their precise roles in anatomical development (Amundson, 2005).

### 5.4. The Trees of Cell Specialization Controlled by Transcription Factors and Epigenetic Enzymes

Cell functionalities, relying on functional cell phenotypes, were developed in a cell colony with the emergence of transcription factors (de Mendoza and Sebé-Pedrós, 2019). Their combinations forming gene regulatory networks (GRNs) may have occurred very early, as many transcription factors were already present as early as 1.5 billion years ago, in LECA, the last eucaryotic common ancestor (de Mendoza and Sebé-Pedrós, 2019). One may assume that, likely due to the necessity to develop functional capabilities to make individual cells able to adapt to changing and often hostile environments, transcription factors have gradually combined into GRNs, constituting the biological support of the expression of functional phenotypes. Furthermore, differentiations are by nature *epigenetic*, insofar as they occur, leading to very different terminal cell types on the basis of the same genome, which naturally creates a role for epigenetic enzymes at the level of chromatin, partly unraveled in Arney and Fisher (2004) in their relationship with transcription factors, and more recently in Atlasi and Stunnenberg (2017).

Such differentiation phenotypes, achieved by specializations or branching points in the trees that, before the emergence of Metazoa 2.0, were likely all reversible, are modules of elementary adaptation to the external environment already present in unicellular constituents:

- germinal or somatic nature (duality germen/soma in sexed reproduction),
- motility or attachment to a matrix,
- emission/reception of (fast or slow) communication between cells of the colony,
- means of absorption of fueling matter and of elimination of toxic residues,
- activator-inhibitor dynamics, leading to space/time periodic behavior of tissues and of intracellular/intercellular signaling pathways, mandatory to maintain continuity of flows in a limited space, and
- friend-or-foe recognition and elimination of (or fight from) foes.

These cell phenotypes, before the closure of the Borromean node, i.e., before the actual emergence of Metazoa 2.0, are still not fixed by epigenetic constraints, and they are thus widely reversible. In other words, the epigenetic landscape is flat. This will change when some newly established differentiation potential (Zhou et al., 2012, 2018), ensured by the cohesion watch hypothesized to be part of the immune system, will force differentiations to become irreversible.

### 5.5. The Working Immune System Involves a Cohesion Watch in Charge of Compatibilities

Indeed, the immune control of cell differentiation should consist firstly of checking their coherence (i.e., that cells follow a coherent differentiation path according to simple rules in terms of the complementary histocompatibility complex—the cohesion watch hypothesized earlier in this construction) and secondly of making these differentiations irreversible. The latter implies the constitution of a potential (Zhou et al., 2012, 2018), or of an entropy, at its highest level in stem cells of the tissue (e.g., hematopoietic stem cells for blood) and at its lowest level in the ultimately differentiated cells of the lineage. Among the differentiated blood cells are lymphocytes, which are in charge of the control of surface antigens of all other tissues.

In more detail, the task of the hypothesized cohesion watch, part of this extended version of the immune system, which must exist already virtually, as inscribed in the self-extracting archive of the genome before fecundation, is thus to ensure compatibilities:

(a) between morphogens of the body plan, able to drive it actually from the zygote in an irreversible way within the 3D space of cells of a given individual (defined by its MHC in vertebrates by some equivalent forerunners in non-vertebrates);
(b) between phenotypic functionalities, ensuring compatibility between differentiation trees that yield lineages within a given subpopulation, and ultimately between cooperating subpopulations (division of work) of terminally differentiated cells; and
(c) between the body plan space distribution and the time distribution of phenotypes in each epigenetic landscape attached to the body plan.

One can think of this cohesion watch as being in charge of irreversibility of differentiations along each tree stemming from the body plan (vertical cohesion), but also of compatibility at each developmental stage between neighboring functionality trees. This involves transversal cohesion, failed for instance in cervical cancer due to histological uncertainty between two different epithelial coverings likely resulting from impaired differentiation of immature renewing cells in one or both lineages, and it is mandatory to form a cohesion net, knitting node after node in all relevant directions.

To mentally illustrate this construction, consider the wickerwork basket. Starting from a circle endowed with lots of connections between its elements, supposed to represent the body plan, functional willow-like twigs stem from each of these elements, representing the great physiological functions of the organism. If no weaving is made between these twigs, the whole set will consist of just flexible differentiation functionalities of a family of cell types, floating freely in the surrounding space unrelated to each other. No cohesion—no division of labor—can result from such unwoven twigs and trees. The task of the cohesion watch is to ensure such weaving during development until tips that are terminally differentiated cells. This naturally includes the solidity of the willow twigs [breaches along the vertical axis resulting in blocked differentiations, as is the case with acute myeloid leukemia (AML)], but the main part of the cohesion watch is to ensure compatibility between (spatially and functionally) neighboring twigs.

Could this hypothesis be tested by evaluation of coherence in the expression of transcription factors responsible for the differentiations of mandatorily compatible tissues at different stages of their differentiations? This could rely on the investigation of intercellular communication means regulating GRNs in different cells, as described in Peter and Davidson (2017) and Erkenbrack et al. (2018).

The emerging capacities of the whole system consisting of the three subsystems, the body plan, trees of phenotypic functionalities giving rise to lineages from virtual pluripotent stem cells and cohesion watch, will now endow the multicellular organism-to-be (after fecundation), in a coherent and stable way. This will consist of making use of division of work and cooperation between the subsystems with functionalities, relying on survival means based on the non-exhaustive list of elementary adaptation phenotypes above and later producing “the great physiological functions” taught to students in medicine and physiology. These capacities will identify a common characteristic of a well-defined species. These might look like the following:

- boundaries with the external environment in both the anatomical spatial and phenotypic (protection) senses,
- strategies to feed on the environment by ingestion of prey,
- friend-or-foe recognition and surveillance against predators,
- abilities to react to hostile environments, whole organism motility (flight) being one such ability,
- integration of all cells by rapid intercellular communication networks,
- reproduction facilities (sexed reproduction by germinal/somatic cell specialization), and
- cognitive processes.

Cognitive processes are indeed among the mandatory functionalities of an evolved multicellular organism (not only vertebrates but including also, for instance, octopuses) under the control of the hypothesized cohesion watch. Conversely, could there exist a support for a possible control of cognitive processes on the immune control of proliferation and differentiation that might explain some inexplicable spontaneous cures of cancer? If so, would the classic immune response (cellular and humoral) be responsible for it, or could it be an effect of the cohesion watch? All physicians are aware of such stories of cures that cannot find any explanation within the corpus of medical knowledge except by a timely intervention of the immune system. An example of a mild one is a plantar wart about to be surgically excised that completely disappeared in one night without any trace on the morning of the intervention; other examples exist for cancer, usually not reported as medical observations, being beyond the scope of contemporary science. This means that, even though the existence of a cohesion watch is primordial for the stability of the organism, it may itself become a part of the organism under the control of superior integrative control, of nervous origin, that unifies a particular individual within a given species with respect to the maintenance of its stability in behavioral life. Michel Jouvet has proposed the interesting hypothesis that the physiological meaning of cortical activity during paradoxical sleep, i.e., dreaming, is a neuronal reprogramming of the individual, a consultation of their genetic program together with their past life personal history, aimed at adapting behavior to be ready to solve issues they will likely meet in their immediate future (Jouvet, 1978, cited by Nathan, 2011).

## 6. Perspectives in Cancer Therapeutics

Within this evolutionary perspective of the design of a multicellular organism, developmental diseases like those mentioned ones above are diseases of the immune system control of the body plan. Assuming a cohesive body plan, which is usually the case, cancer appears as a loss of control of the immune system on the trees of differentiations and on compatibility connections between them. Cancer may thus be the result of flaws in the means of control or the result of incoherence in the control subsystem itself. Auto-immune diseases are clearly due to incoherence in this controlling immune subsystem.

From a cancer therapeutic viewpoint, as stated by Lineweaver et al. (2014), attacking cancer by blocking its proliferation using chemotherapies or radiotherapies is clearly a short-sighted method. It may work completely in some cases, though most often partially and temporarily, but as long as the epigenetic system of control on differentiations fails, the dynamics of cancer will prevail. This may be avoided if the immune response keeps residual cancer cells in check, preventing them from excessive proliferation; this is usually called cancer dormancy and is not clinically distinguishable from a cure if it is indefinitely prolonged.

Despite this limitation, relying on the existing cell-killing therapies, which may be (cytotoxic) chemotherapies, (cytostatic) targeted therapies or immunotherapies, mathematical models have been developed with corresponding theoretically optimized treatment strategies representing monotherapies or more successfully combination therapies in cancer (Pouchol, 2018; Pouchol et al., 2018; Jarrett et al., 2020). The starting point of this article was a solution that—inscribed in the time scale of a human life and not in the billion-year perspective presented above—aims to be immediately useful in the clinic. Taking advantage only of what we know presently of the behavior of cancer cells exposed to cytotoxic and cytostatic drugs in the framework of a cell population and not of the history of their making, as is the goal of the presently proposed billion-year perspective for therapeutics, this has been briefly described in the first section of the present study. Among modern immunotherapies, immune checkpoint inhibitors (ICIs), by boosting the immune response by lymphocytes that attack tumor cells, e.g., in the case of melanoma treated with the combination ipilimumab + nivolumab, may be successful with about 60% of objective response rates in patients, of which 20% of total cases can even reach complete long remissions. Unfortunately, there may also more rarely exist total failures that result in non-responders in 30% of cases and even in so-called hyperprogressors (i.e., experiencing accelerated tumor growth defined by at least a 2-fold tumor growth rate increase compared with pre-immunotherapy rate) in the remaining 10% (Márquez-Rodas et al., 2015; Frelaut et al., 2019; Liu et al., 2019). Such cell-killing strategies may be successful by mending a breach in the control of cell proliferation, but if a fragility remains in the control of differentiations somewhere in the organism, a relapse may occur, possibly with cells that will have been selected for their robustness and will be less sensitive to the treatment.

This should induce us to enhance our understanding of the role of the immune system (and more precisely of the cohesion watch) in the hypothesized Borromean system upon which relies a physiologically well-constituted animal. Rather than fighting uncontrolled proliferation, could we repair altered control on differentiations? Cell-killing strategies, whether they rely on chemotherapies or on modern immune cell-enhancing drugs, miss the basic targets, which are differentiation sites. There are possibly only two known successful non-cell-killing therapies: imatinib in chronic myelogenous leukemia (CML) (Hochhaus et al., 2008), where imatinib [or drugs of the same family of tyrosine kinase inhibitors (TKIs)] blocks the ATP pocket of a chimeric protein, BCR-ABL, which itself is due to a fusion of genes, normalizing proliferation; secondly, there is all-trans retinoic acid (ATRA) in acute promyelocytic leukemia (APL = AML3 in the old French-American-British, FAB, classification of acute myeloid leukemias) (Haferlach, 2008), where ATRA degrades the PML-RARα chimeric protein (that also results from a fusion of genes) that blocks maturation of the myeloid lineage at the promyelocytic stage. Many redifferentiation strategies close to this one have been attempted, but all the others have failed.

Nevertheless, this could be the future of cancer therapeutics: intervention at the differentiation sites on transcription factors or on factors that control them, i.e., enforcing the cohesion watch connection rather than killing cheater cells; in other words, the solution could be in mending a net with a hole in it rather than trying to kill sharks that have escaped containment. Alternatively, this goal can be illustrated with a sociological metaphor. This is indeed relevant as, in the hierarchy of levels of organization that goes from genes to cells and from cells to multicellular organisms, the next level is evolving societies of living multicellular individuals. In light of the above, rather than killing cheater cells through cannonade (i.e., by chemotherapies) or by enforcing the aggressiveness of the police (i.e., by immune checkpoint inhibitors), would it not be better to assess how we enforce the law? The law here is the cohesion watch that exists as a plan in the genome before embodiment in development and later as an acellular communication network between tissues and organs. This could be done by repairing broken local social bonds between functionalities (expressed after embodiment as tissues and organs), as neither the army nor the police are the best means to establish harmonious working links of cooperation between citizens. Citizens in multicellular organisms are the somatic cells in tissues and organs that are normally organized toward a common goal: preservation of the genome toward reproduction, and to that purpose, the preservation of the health of the global society of cells. To be able to do this, a better understanding of the mechanisms of control of differentiation at the level of local transcription factors and at the level of chromatin is needed. The development of epigenetic drugs is promising, widely relying on inhibitors of DNA methyltransferases (iDNMTs) or of histone deacetylases (iHDACs) (Roberti et al., 2019). These could be a starting point, provided that the interactions between epigenetic enzymes and transcription factors can be unraveled (Arney and Fisher, 2004). This could lead to future differentiation-repairing cancer therapies that would be precisely targeted at the best possible sites of multicellular organisms and would disregard cell-killing therapies, except to accelerate a clearance process, as with ATRA, which is usually delivered together with an anthracyclin, resulting in a complete cure of APL (Haferlach, 2008), in a remote past of cancer medicine. Another route to explore might be to examine, following Davidson's works on intercellular communication means that regulate consistency between intracellular GRNs during development (Peter and Davidson, 2017; Erkenbrack et al., 2018), targets and reestablish such impaired intercellular signaling.

## 7. Conclusion

Moving away from deliberations on the evolution of a cell population at the time scale of a human, which is nevertheless undoubtedly if high interest in therapeutics, an example of which is that what this article advocated along with Robert Gatenby and his colleagues at the Moffitt Cancer Center in Tampa (Gatenby et al., 2009; Gillies et al., 2012; West et al., 2020) in terms of mathematical models designed to optimize strategies based on combined cell-killing therapies (Pouchol, 2018; Pouchol et al., 2018; Jarrett et al., 2020), this article further presents an evolutionary point of view on cancer from a billion-year perspective that, from questions on plasticity in cancer, has guided the development of ideas resorting to what is now named the *philosophy of cancer* (Pradeu, 2012, 2019; Bertolaso, 2016; Laplane, 2016; Plutynski, 2018). The view takes basis in various philosophers of cancer, walking a long and winding path toward a fundamental understanding of multicellularity and of its alterations in cancer. Ultimately, following this path should lead to correct impaired control of differentiation rather than, or at least together with, control of proliferation. Much of what is presented here, as much as it is possible to rely on published observations or opinions, is of speculative nature, in particular with respect to the exploration, discovery and generalization of non-cell-killing therapies, which so far remain elusive in the clinic. Nevertheless, in a time when humanities, mathematics, biology and medicine are uniting their efforts to overcome the struggle against cancer, this approach is hopefully a timely one.

## Author Contributions

The author confirms being the sole contributor of this work and has approved it for publication.

## Conflict of Interest

The author declares that the research was conducted in the absence of any commercial or financial relationships that could be construed as a potential conflict of interest.
